# Pharmacological modulation of ventral tegmental area neurons elicits changes in trigeminovascular sensory processing and is accompanied by glycemic changes: Implications for migraine

**DOI:** 10.1177/03331024221110111

**Published:** 2022-10-18

**Authors:** Margarida Martins-Oliveira, Simon Akerman, Philip R Holland, Isaura Tavares, Peter J Goadsby

**Affiliations:** 1 Headache Group, Wolfson Centre for Age-Related Disease, Institute of Psychiatry, Psychology and Neuroscience, King's College London, UK; 2 Department of Nutrition and Metabolism, NOVA Medical School|Faculdade de Ciências Médicas, NMS|FCM, Universidade Nova de Lisboa; Lisboa, Portugal; 3 Department of Biomedicine, Faculty of Medicine of University of Porto, Porto, Portugal; 4 Institute of Investigation and Innovation in Health (i3S), University of Porto, Porto, Portugal; 5 Department of Neural and Pain Sciences, University of Maryland Baltimore, Baltimore, Maryland, USA; 6 Department of Neurology, University of California, Los Angeles, Los Angeles CA USA

**Keywords:** Ventral tegmental area, migraine, nociception, blood glucose, naratriptan, PACAP

## Abstract

**Background:**

Imaging migraine premonitory studies show increased midbrain activation consistent with the ventral tegmental area, an area involved in pain modulation and hedonic feeding. We investigated ventral tegmental area pharmacological modulation effects on trigeminovascular processing and consequent glycemic levels, which could be involved in appetite changes in susceptible migraine patients.

**Methods:**

Serotonin and pituitary adenylate cyclase-activating polypeptide receptors immunohistochemistry was performed in ventral tegmental area parabrachial pigmented nucleus of male Sprague Dawley rats. *In vivo* trigeminocervical complex neuronal responses to dura mater nociceptive electrical stimulation, and facial mechanical stimulation of the ophthalmic dermatome were recorded. Changes in trigeminocervical complex responses following ventral tegmental area parabrachial pigmented nucleus microinjection of glutamate, bicuculline, naratriptan, pituitary adenylate cyclase-activating polypeptide-38 and quinpirole were measured, and blood glucose levels assessed pre- and post-microinjection.

**Results:**

Glutamatergic stimulation of ventral tegmental area parabrachial pigmented nucleus neurons reduced nociceptive and spontaneous trigeminocervical complex neuronal firing. Naratriptan, pituitary adenylate cyclase-activating polypeptide-38 and quinpirole inhibited trigeminovascular spontaneous activity, and trigeminocervical complex neuronal responses to dural-evoked electrical and mechanical noxious stimulation. Trigeminovascular sensory processing through modulation of the ventral tegmental area parabrachial pigmented nucleus resulted in reduced circulating glucose levels.

**Conclusion:**

Pharmacological modulation of ventral tegmental area parabrachial pigmented nucleus neurons elicits changes in trigeminovascular sensory processing. The interplay between ventral tegmental area parabrachial pigmented nucleus activity and the sensory processing by the trigeminovascular system may be relevant to understand associated sensory and homeostatic symptoms in susceptible migraine patients.

## Introduction

Human imaging studies have shown that several areas of the brain (e.g. hypothalamus, ventral tegmental area (VTA), nucleus accumbens (NAc)) are active during the premonitory phase of spontaneous migraine (1,2), and in nitroglycerin-induced migraine attacks (3). The midbrain VTA is involved in goal-directed behavior and in processing for natural rewards (e.g. food reward), alcohol and drugs of abuse, and is further linked to pain modulation (4–7). Recently, a connection between the periaqueductal grey (PAG) matter and the VTA has shown to mediate aversive behavior in an inflammatory model of headache (8).

The VTA is a heterogeneous nucleus containing dopaminergic (DA) neurons (≈65%) with reciprocal projections to several nuclei including the NAc, prefrontal cortex, amygdala, hypothalamus and periaqueductal gray (PAG) (9). It also contains GABAergic (≈30%) and glutamatergic (GLU) neurons (≈5%) (10–13), that regulate local VTA DA neuronal activity, but also have projection targets similar to those of DA neurons (14–19). It exhibits antero-posterior anatomical and functional heterogeneity with topographic neuronal projections (6,20). Specifically, the parabrachial pigmented nucleus of the VTA (VTA^PBP^) (21,22), sends neuronal projections to the medial prefrontal cortex (mPFC), and medial and lateral shell of the NAc (23–26). We hypothesized that modulation of the VTA may influence trigeminovascular processing, which could be involved in appetite changes in susceptible migraine patients (27,28). We used an animal model of acute dural nociceptive activation of the trigeminovascular system, which has reliably predicted clinical efficacy of migraine therapeutics (29). The literature reports the location of migraine-relevant 5-HT_1_ and pituitary adenylate cyclase activating polypeptide (PACAP) receptors within the VTA (by using *in situ* hybridization (30), quantitative autoradiographic mapping (31) or by immunohistochemistry and by double immunofluorescence (32)) but was inconsistent about the location specifically within the VTA subnuclei, namely the VTA^PBP^. Hence, we first used immunofluorescence to perform a topographic evaluation of the expression of 5-HT_1B_, 5-HT_1D_, PAC_1_, VPAC_1_ and VPAC_2_ receptors in the VTA^PBP^ and to guide pharmacological administration. We then studied the effects of the modulation of excitatory and inhibitory inputs within the VTA^PBP^ on trigeminovascular neuronal responses by manipulating VTA^PBP^ neurons with glutamate and a competitive antagonist of GABA_A_ receptors. We further investigated potential therapeutic modulation by microinjecting naratriptan, a 5-HT_1B/D/F_ receptor agonist widely used in migraine treatment; PACAP38, a peptide that, when administered intravenously, induces delayed migraine-like headaches in susceptible migraine patients (33); and a DA D_2_/D_3_ receptor agonist, known to inhibit trigeminovascular responses in animals (34). Since trigeminal cell activation has been associated with blood glucose changes *in vivo* (28,35), we further explored whether peripheral glucose levels were influenced by trigeminovascular sensory processing through modulation of VTA^PBP^ neurons. Preliminary results have been previously presented (36,37).

## Materials and methods

All experiments were conducted in agreement with the guidelines of the Institutional Animal Care and Use Committee (University of California, San Francisco), the UK Home Office Animals (Scientific Procedures) Act 1986, the ARRIVE guidelines (38) and the Committee for Research and Ethical Issues of International Association for the Study of Pain (39). Male Sprague Dawley rats were group-housed and maintained under standard conditions (12 h light–dark cycles; lights ON at 07:00) with food and water available *ad libitum*. The subjective bias when allocating the animals to the experimental groups was minimized by arbitrarily housing the animals in pairs upon their arrival, then the animals were randomly picked from the cage for each procedure. To avoid confounding effects regarding the diurnal cycle, all experiments initiated between 8.00–9.00 am.

### Immunohistofluorescence characterization of the VTA^PBP^

Animals (315–330 g, *n* = 4; Charles River, France) were euthanized with pentobarbital sodium and perfused with 250 ml of heparinized phosphate-buffered saline through the ascending aorta, followed by 300 ml of a fixative solution containing 4% paraformaldehyde. The brain was removed and fixated (35). Serial coronal sections (30 μm-thick) containing the VTA^PBP^, hypothalamus or PAG were cut on a cryostat and processed. Staining was visualized using a fluorescence microscope coupled to digital camera and image software (AxioVision 4.8.2, Carl Zeiss). Briefly, sections were washed in phosphate buffered saline (PBS) and after 60 min incubation in a blocking solution of PBS, 5% normal goat serum (Sigma-Aldrich), and 0.25% Triton x-100 (Alfa Aesar), sections were incubated overnight at 4°C with primary antibody specific to 5-HT_1B_ or 5-HT_1D_ receptors (1:75; ASR-022; ASR-023; Alomone Labs). Sections were washed and incubated in goat anti-rabbit Fluorescein for 90 min (1:250; FI-1000, Vector Laboratories). Another set of sections were washed in PBS, incubated for 30 minutes in PBS with 1% sodium borohydride (Sigma-Aldrich) and washed in PBS. After 60 min incubation in a blocking solution of PBS, 5% normal goat or horse serum (Sigma-Aldrich), and 0.25% Triton x-100, sections were incubated overnight at 4°C with primary antibody specific to PAC_1_, VPAC_1_ and VPAC_2_ receptors (1:100; AVR-003; AVR-001; AVR-002; Alomone Labs), made up in PBS, 2% normal goat or horse serum (Vector Laboratories), and 0.25% Triton x-100. Sections were washed and incubated for 90 min in donkey anti-rabbit Alexa Fluor 594 (for PAC_1_ and VPAC_2_, 1:1000, Life Technologies Corporation) or goat anti-rabbit Texas Red (for VPAC_1_, 1:1000, TI-1000, Vector Laboratories). Sections were then mounted with DAPI. Primary antibodies were chosen based on recent published studies (40–43) and the specificity was demonstrated by the suppliers. Additionally, samples were processed with the omission of primary antibodies, and the staining of brain areas known to contain each receptor was performed.

### In vivo electrophysiology

The surgical preparation and recording setup has been detailed previously (44). Briefly, animals (270–340 g, *n* = 70, Charles River, USA or UK) were anesthetized with 5% (v/v) isoflurane (induction) and propofol solution (maintenance). Body temperature, respiratory rate, end-tidal CO_2_ and blood pressure were continuously monitored. After fixation of the skull in a stereotaxic frame rats were ventilated with oxygen-enriched air.

To access the dura mater and middle meningeal artery (MMA), a craniotomy was performed with a saline-cooled drill and the underlying dura mater covered in mineral oil. To access the TCC, muscles of the dorsal neck were separated, a cervical (C1) laminectomy performed, and the dura mater incised to expose the caudal medulla oblongata. A piezo-electric microelectrode positioner was used to locate the optimal recording site within the TCC. Animals were left to stabilize for at least 60 min before recordings.

**Figure 1.**
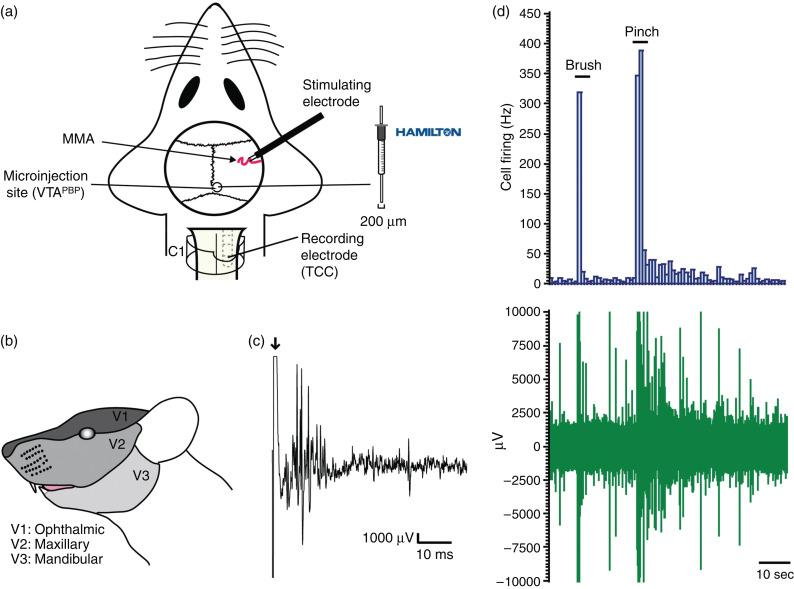
Overview of the experimental setup and neuronal characteristics. (a) Experimental setup with dural electrical stimulation, recording of neurons in the TCC and VTA^PBP^ microinjections. (b) All neurons studied were wide dynamic range (WDR); responsive to both noxious and innocuous stimulation, with cutaneous receptive field in the first (V1; ophthalmic) division of the trigeminal nerve. (c) An original tracing from a typical unit (second-order neuron) responding to electrical stimulation of the dura mater adjacent to the MMA (latencies in the Aδ-fiber range). Black arrow represents stimulus artefact and (d) Original example of the electrophysiological neuronal response to innocuous brush and noxious pinch of the cutaneous V1 receptive field. Bottom panel is original electrophysiological output, top panel is responses that cross the window discriminator. C1, spinal cord cervical 1; MMA, middle meningeal artery; TCC, trigeminocervical complex; VTA^PBP^, parabrachial pigmented nucleus of the ventral tegmental area.

A bipolar stimulating electrode connected to a stimulus isolation unit was placed on the intact dura mater adjacent to the MMA for electrical stimulation of the perivascular afferents of the trigeminal nerve (Figure 1A). Stimulation of primary trigeminal afferents was performed with supramaximally square wave pulses generated by a Grass S88 stimulator. Dural nociceptive neurons in the TCC were identified via electrical stimuli (9–15 V, 0.15–0.3 ms, 0.4–0.5 Hz, 20 sweeps), activating trigeminal Aδ-fibers with approximate latencies between 4–20 ms. By using low stimulation parameters, we activated only Aδ-fibers.

Extracellular recordings were made from wide dynamic range (WDR) neurons in the TCC, activated by dural stimulation using tungsten microelectrodes. Neurons were characterized for their cutaneous and deep receptive fields. The cutaneous receptive field was assessed in all three territories of the trigeminal innervation (Figure 1B) and was identified as the recording electrode was advanced in the spinal cord. The receptive field was assessed for both innocuous (gentle brush), and noxious inputs (pinch) (Figure 1D). When a neuron sensitive to stimulation of the ophthalmic (V1) dermatome of the trigeminal nerve was identified it was tested for convergent input from the dura mater.

### Drugs and microinjections

Animals were microinjected unilaterally into the VTA^PBP^, ipsilateral to the recording site in the TCC, with glutamate (50 mM; Sigma-Aldrich); bicuculline (2 mM; Tocris Bioscience); naratriptan (25 mM; Sigma-Aldrich); pituitary adenylate cyclase activating polypeptide (PACAP38; 100 μM; Tocris Bioscience); quinpirole (4 μg in 400 nL; Sigma-Aldrich); or saline as vehicle control. All drugs were dissolved in sterile saline solution (0.9% sodium chloride, Baxter International Inc.). On the day of the experiment, drugs were dissolved in 2% of dye (Chicago Sky Blue 6B, Alfa Aesar), except for naratriptan and quinpirole due to dissolving difficulties. Volumes were given in a range of 300–400 nL. Doses were chosen based on previous studies (45–47). An area of bone directly above the coordinates of the VTA^PBP^ was thinned and removed and the dura mater pierced to allow entry of a microliter syringe (Figure 1A). The VTA^PBP^ stereotaxic coordinates were: from bregma, AP 5.3 mm; ML ±0.7 mm; DV-7.5 mm from dura mater (22). The microinjection sites were either marked by deposition of dye or by the syringe track with blood (naratriptan and quinpirole administration). We also performed preliminary studies to confirm there was no spread of the dye solution outside the target area (data not shown). The location of the injection and dye was restricted to the target area of VTA^PBP^ in a longitudinal manner and did not reach the posterior hypothalamus (anterior to the VTA^PBP^) or the substantia nigra compacta (lateral to the VTA^PBP^).

### Blood glucose levels

Tail vein blood glucose was quantified using a glucometer (FreeStyle Lite, Abbott Diabetes Care Inc). Given that a non-diabetic animal was used and there was no indication of VTA direct effects on glycemia, single time-point blood glucose measurements were performed, as reported in clinical (48,49) and rodent (35) migraine studies. To confirm a normoglycemic state, animals were assessed after induction anesthesia and before surgical preparation. Blood glucose levels were then quantified at two specific time-points: before inserting the recording electrode and the microinjection syringe into the brain, and at 60 min post-microinjection, as described (35).

### Postsurgical examination of tissue

Animals were euthanized and an electrothermolytic lesion was made in the TCC. Brains and spinal cord were removed and sliced (60-μm and 40 µm-thick coronal sections) on a freezing cryostat. The exact microinjection sites were verified using a light microscope (Axioplan Microscope; Carl Zeiss) and the rat brain atlas (22).

### Experimental design and statistical analysis

Trains of 20 stimuli were delivered at 5 min intervals to assess the baseline response to dural electrical stimulation. Data collected for Aδ-fibers represent the normalized data for the number of cells firing over a 10 ms period in the region 4–20 ms post-stimulation over the 20 sweeps. Responses were analyzed using post-stimulus histograms with a sweep length of 100 ms and a bin width of 1 ms that separated Aδ-fiber-activated firing. When stable baseline values of the dural-evoked responses were achieved and cutaneous and deep receptive field inputs from the ophthalmic division of the trigeminal nerve were obtained, responses were tested for up to 60 min following microinjections. Ongoing spontaneous trigeminal neuronal activity (spikes/second, Hz) was recorded continuously, and data related to 120–150 sec preceding the dural stimulation using peri-stimulus histograms was analyzed, as described (50). This activity was analyzed as cumulative rate histograms in which neuronal activity gated through the amplitude discriminator was collected into successive bins.

Two types of mechanical stimuli were delivered to the ipsilateral ophthalmic dermatome of the trigeminal nerve: 1) innocuous brushing and 2) noxious pinching, as described previously (50) Responses were recorded immediately before, and 15, 30 and 60 min after microinjection. Only one baseline for the mechanical stimuli was taken to avoid sensitization prior to drug administration. Spontaneous discharges were documented for 5 sec after application of the stimulus and the mean firing rate (Hz) response to each mechanical stimulus was analyzed. Data is expressed as mean ± SEM and the mean firing rate upon application of each stimulus prior to drug microinjection was taken to be 100%. Using previous experience (35), a minimum of seven animals were used in electrophysiological studies to measure time points up to 60 min.

Statistical analysis was performed in raw data using IBM SPSS (v23.0). To detect whether there was a significant effect over time (pre-injection and eight individual time point values at 5, 10, 15, 20, 25, 30, 45 and 60 min post-injection) we used one-way ANOVA for repeated measures (RM-ANOVA) with Bonferroni *post-hoc* correction for multiple comparisons, using a 95% confidence interval. If Mauchly’s test of sphericity was violated, appropriate corrections to the degrees of freedom were made according to Greenhouse–Geisser (51). Student’s paired *t*-test (two-tailed) was used for *post-hoc* analysis of individual time points comparing to pre-injection values. To compare the treatment group (bicuculline) with the vehicle treatment group we used two-way repeated measures ANOVA with a Greenhouse-Geisser correction and Tukey’s test for post-hoc analysis. Statistical significance was set at *P* < 0.05 level. The experimenter was aware of (not blinded to) each step of the experimental process.

## Results

### Serotonin and PACAP receptors are present in the VTA^PBP^

**Figure 2.**
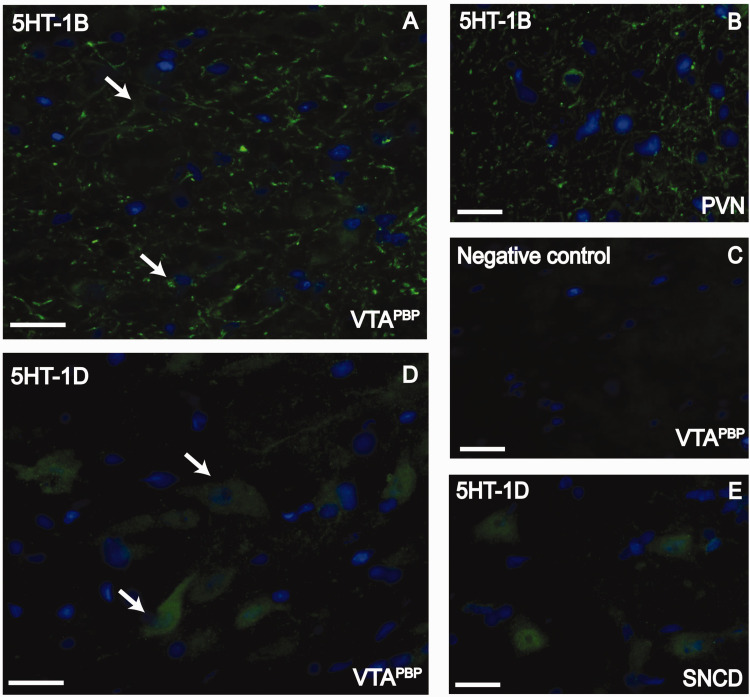
Immunofluorescence staining for 5HT_1B_ and 5HT_1D_ receptors in the VTA^PBP^. (a) 5HT_1B_ receptors in the VTA^PBP^ (green); (b) 5HT_1B_ receptors in the paraventricular nucleus of the hypothalamus (PVN), as positive control (green); (c) Negative control in the VTA^PBP^, obtained by omitting the primary antibodies; (d) 5HT_1D_ receptors in the VTA^PBP^ (green) and (e) 5HT_1D_ receptors in the substantia nigra, compact part, dorsal tier (SNCD) (green), as a positive control; Receptors are colocalized with DAPI (blue, nuclear marker). Arrows indicate examples in each image. Scale bars, 20 μm.

**Figure 3.**
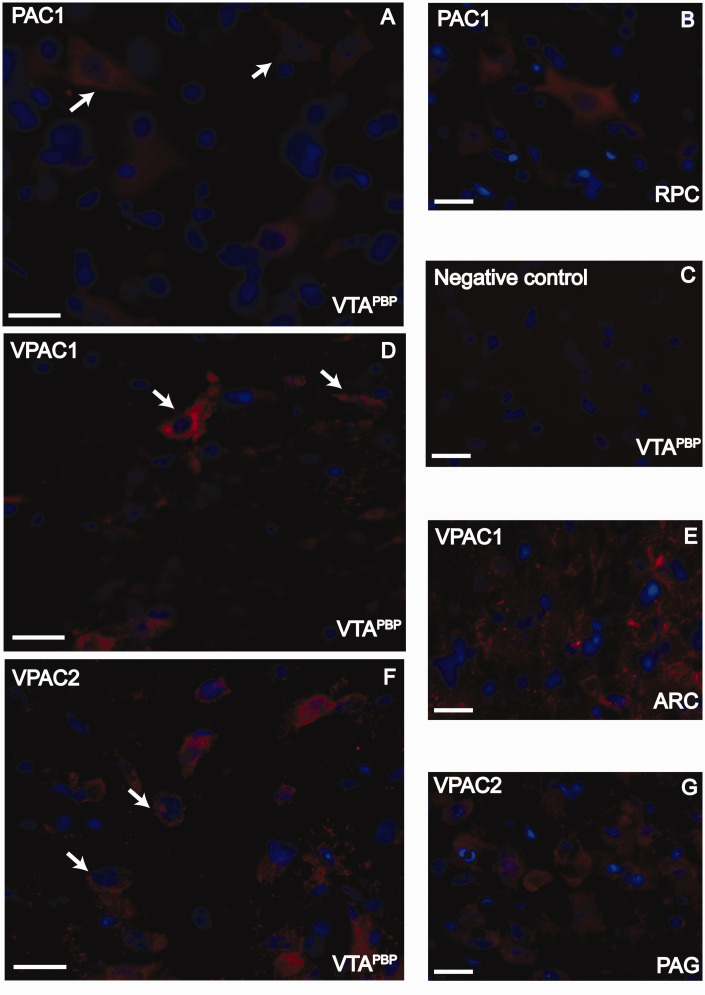
Immunofluorescence staining for PAC_1_, VPAC_1_ and VPAC_2_ receptors in the VTA^PBP^. (a) PAC_1_ receptors in the VTA^PBP^ (red); (b) PAC_1_ receptors in the red nucleus, parvicellular part (RPC), as a positive control (red); (c) Negative control in the VTA^PBP^, obtained by omitting the primary antibodies. (d) VPAC_1_ receptors in the VTA^PBP^ (red); (e) VPAC_1_ receptors in the arcuate nucleus of the hypothalamus (ARC), as a positive control (red); (f) VPAC_2_ receptors in the VTA^PBP^ (red) and (g) VPAC_2_ receptors in the periaqueductal gray (PAG), as a positive control (red); Receptors are colocalized with DAPI (blue, nuclear marker). Arrows indicate examples in each image. Scale bars, 20 μm.

Given that antibodies can be nonspecific, we assume a putative expression herein. Putative 5-HT_1B_ and 5-HT_1D_ receptors (green labelling) were present within the VTA^PBP^, with 5-HT_1B_ receptors more visible associated with fibers, whereas 5-HT_1D_ receptors with cell bodies (Figure 2A, 2D). These receptors were also present in the paraventricular nucleus of the hypothalamus (PVN), and the substantia nigra, compact part, dorsal tier (SNCD) (Figure 2B, 2E). Putative receptors for PACAP, namely PAC_1_, VPAC_1_ and VPAC_2_ receptors (red 1abelling), were distributed throughout all levels of the VTA^PBP^ region (Figure 3A, 3D, 3F). These receptors were also present in other brain structures: the red nucleus, parvicellular part (RPC), the arcuate nucleus of the hypothalamus (ARC) and the PAG (Figure 3B, 3E, 3G). All receptors were colocalized with DAPI (nuclear marker; blue), indicating its presence in cell bodies. No specific staining was observed when the primary antibody was omitted (Figure 2C and Figure 3C).

### In vivo electrophysiology experiments

#### Physiology parameters, electrophysiological data, and postsurgical histology

A total of 49 animals had microinjections inside the VTA^PBP^, a mean body weight of 301 ± 2 g and blood glucose levels within physiological levels at the beginning of the experiment (6.33 ± 0.06 mmol/L). Data from animals in which histological analysis showed microinjection placements outside the VTA^PBP^ (*n* = 21) were excluded from the main analysis.

Extracellular recordings in the TCC were made from a total of 54 neurons. In animals that received a second microinjection (*n* = 4, vehicle control; *n* = 1, bicuculline) there was a washout period of 90–120 min. Neurons responding to dural electrical stimulation responded with an average latency of 10.6 ± 0.2 ms (range 4–20 ms, Figure 1C shows an example of evoked neuronal firing) and were classified as Aδ-fibers. Very few C-fiber latency (beyond 20 ms) responses were observed, and therefore we were only able to quantify Aδ-fiber responses. Most neurons were in lamina V of the dorsal horn of the cervicomedullary junction, with 13 neurons in lamina II-IV, at an average depth of 588 ± 23 µm and the electrode placement was confirmed in all animals (Figure S1A and S1B). The mean ongoing spontaneous firing rate pre-injection (baseline) was 28 ± 2.3 Hz (range 1.8–64.8 Hz), with most neurons responding between 5 and 25 Hz; this is within the same range as previously demonstrated (44). The location of microinjections inside the VTA^PBP^ (black dots) and adjacent to the VTA^PBP^ (black stars) are indicated in representative atlas plate rat brain sections (22) (Figure S1C) and histological example in Figure S1D.

#### Glutamatergic stimulation of the VTA^PBP^ reduces nociceptive and spontaneous TCC neuronal firing

**Figure 4.**
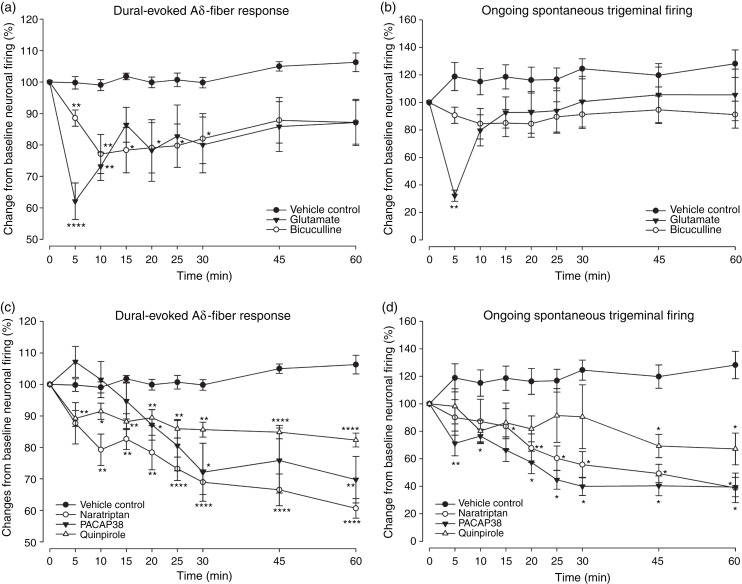
Effects of pharmacological manipulation of VTA^PBP^ on dural-evoked and spontaneous neuronal firing in the trigeminocervical complex (TCC). (a) Time course changes in the average response of dural-evoked Aδ-fiber trigeminal neuronal firing following microinjection of glutamate (*n* = 8) and bicuculline (*n* = 11), which significantly decreased neuronal responses by a maximum of 38% and 23%, respectively. (b) Time course of ongoing spontaneous trigeminal neuronal firing in response to glutamate (*n* = 8), which significantly decreased neuronal responses by a maximum of 68% at 5 min and then recovered, and bicuculline (*n* = 11), which had no significant effect compared to pre-injection levels. (c) Time course of the average response of intracranial dural-evoked Aδ-fiber trigeminal neuronal firing following microinjection of naratriptan (*n* = 8), PACAP38 (*n* = 7) and quinpirole (*n* = 8), which significantly decreased neuronal responses by a maximum of 39%, 30% and 18%, respectively and (d) Time course of spontaneous trigeminal neuronal firing in response to naratriptan (*n* = 8), PACAP38 (*n* = 7) and quinpirole (*n* = 8), which significantly decreased neuronal responses by a maximum of 61%, 60% and 33%, respectively. In all panels vehicle control (*n* = 12) had no significant effects of neuronal responses. Data have been normalized to represent the percentage change from baseline, and are expressed as means ± SEM. *P < 0.05; **P < 0.01; ***P < 0.001; ****P < 0.0001.

Glutamate significantly inhibited dural-evoked responses (*F*_2.3,16.3_ =4.615; *P* = 0.022; *n* = 8) (Figure 4A, S2A) and spontaneous neuronal firing (*F*_2.1,15.3_ = 8.372; P = 0.003; *n* = 8) (Figure 4B). Moreover, bicuculline significantly reduced dural-evoked neuronal firing within the TCC (*F*_2.4,24.6_ = 3.791; *P* = 0.029; *n* = 11) (Figure 4A, S2A), however had no significant effect on the ongoing spontaneous activity when compared to pre-injection (*F*_2.8,28.2_ = 1.229; *P* = 0.316; *n* = 11) (Figure 4B). Microinjection of vehicle control in the VTA^PBP^ had no significant effect on Aδ-fiber responses (*F*_4.2,46_ = 2.030; *P* = 0.102; *n* = 12) (Figure 4A, S2A) and on ongoing spontaneous activity (*F*_3.5,39_ = 2.596; *P* = 0.056; *n* = 12) of trigeminal second-order neurons (Figure 4B). Given that the bicuculline treatment group showed greater variance in ongoing spontaneous responses, we aimed to confirm whether the larger variance may have affected the meaning of the results (one-way RM-ANOVA did not show significance throughout the 60 min experiment). We further compared spontaneous firing responses between vehicle control and bicuculline treatment and a two-way RM- ANOVA revealed that the main effect of treatment group on the average spontaneous firing responses across time was not statistically significant (F_1,21_ = 2.958, *p* = 0.1).

A summary of ongoing spontaneous firing rate after 5 and 10 min for each treatment group is presented in Table S1.

#### Naratriptan and PACAP38 modulation of VTA^PBP^ inhibits nociceptive and spontaneous TCC neuronal firing

Microinjection of naratriptan into the VTA^PBP^ significantly reduced dural-evoked neuronal firing within the TCC (*F*_2.9,20.7_ = 12.163; *P* = 0.000; *n* = 8) (Figure 4C, S2B) and had a significant inhibitory effect on the ongoing spontaneous activity (*F*_2.0,14.2_ = 5.616; *P* = 0.015; *n* = 8) (Figure 4D). Similarly, PACAP38 significantly reduced dural-evoked neuronal firing within the TCC (*F*_1.7,10.6_ = 9.392; *P* = 0.005; *n* = 7) (Figure 4C, S2B), and ongoing spontaneous activity (*F*_1.3,8.1_ =7.872; *P* = 0.017; *n* = 7) (Figure 4D). Moreover, quinpirole significantly reduced dural-evoked neuronal firing within the TCC (*F*_3.6,25.8_ = 7.005; *P* = 0.001; *n* = 8) (Figure 4C, S2B), and ongoing spontaneous activity (*F*_3.1,21.9_ = 3.491; *P* = 0.031; *n* = 8) (Figure 4D). A summary of ongoing spontaneous firing rate after 5 and 10 min for each treatment group is presented in Table S1.

#### Naratriptan and PACAP38 modulation of VTA^PBP^ reduced innocuous and noxious facial TCC responses

**Figure 5.**
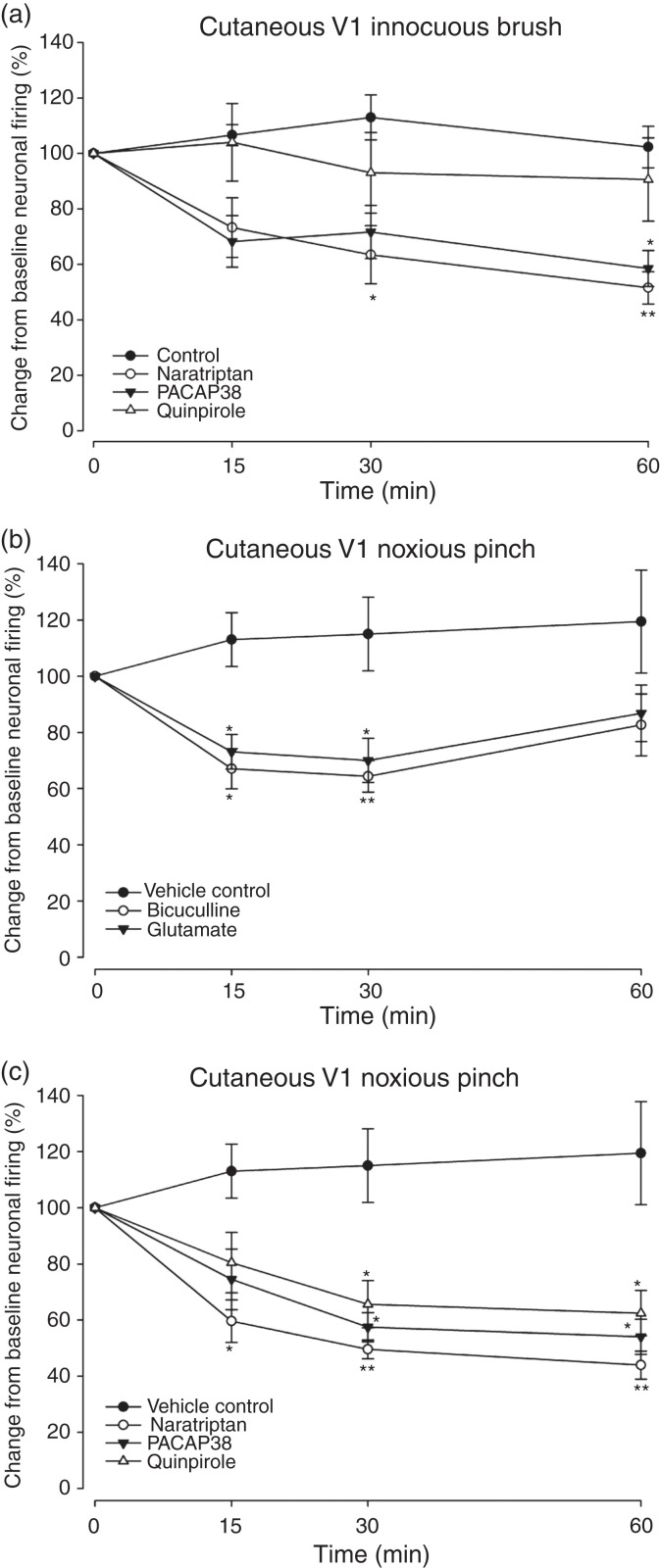
Effect of VTA^PBP^ pharmacological modulation on neuronal firing in the trigeminocervical complex (TCC), in response to mechanical stimulation of the ophthalmic dermatome (V1). Time course changes in the average response of TCC neurons to somatosensory-evoked stimulation of the cutaneous facial receptive field. (a) response to innocuous brush stimulation following microinjection of naratriptan (*n* = 8), PACAP38 (*n* = 7) and quinpirole (*n* = 8) into the VTA^PBP^; (b) response to noxious pinch stimulation following microinjection of glutamate (*n* = 7) and bicuculline (*n* = 4) into the VTA^PBP^; and (c) response to noxious pinch stimulation following microinjection of naratriptan (*n* = 8), PACAP38 (*n* = 7) and quinpirole (*n* = 8) into the VTA^PBP^. In all panels vehicle control (*n* = 11) had no significant effects of neuronal responses. Data have been normalized to represent the percentage change from baseline, and are expressed as means ± SEM. *p < 0.05; **p < 0.01.

Measurements of neuronal responses to cutaneous mechanical stimulation of the ophthalmic dermatome were made from 45 neurons. Data from the recordings of nine neurons were excluded from this analysis due to incomplete data collection for one or more time points of the experimental design (*n* = 1, vehicle control; *n* = 7, bicuculline; *n* = 1, glutamate). Vehicle control had no significant effect on responses to either innocuous brush (*F*_3,30_ = 0.964; *P* = 0.422; *n* = 11) (Figure 5A) or noxious pinch (*F*_3,30_ = 0.607; *P* = 0.615; *n* = 11) (Figure 5B) of cutaneous facial receptive fields. For both glutamate and bicuculline, there were no effects in response to innocuous brush (Glu: *F*_3,18_ = 1.037; *P* = 0.400; *n* = 7; bicuculline: *F*_3,9_ = 1.895; *P* = 0.201; *n* = 4). Both significantly reduced responses to noxious pinch (Glu: *F*_3,18_ = 5.132; *P* = 0.010; *n* = 7; bicuculline: *F*_3,9_ =12.169; *P* = 0.002; *n* = 4) (Figure 5B). Naratriptan significantly inhibited neuronal responses to innocuous brush (*F*_3,21_ = 7.516; *P* = 0.001; *n* = 8) (Figure 5A) and noxious pinch (*F*_1.5,10.8_ = 12.602; P = 0.002; *n* = 8) (Figure 5C). Similarly, PACAP38 significantly inhibited responses to innocuous brush (*F*_3,18_ = 5.248; *P* = 0.009; *n* = 7) (Figure 5A) and noxious pinch (*F*_1.1,6.9_ = 5.622; *P* = 0.047; *n* = 7) (Figure 5C). Furthermore, quinpirole did not affect innocuous responses (*F*_3,21_ = 0.587; *P* = 0.630; *n* = 8) (Figure 5A), but significantly inhibited responses to noxious pinch (*F*_1.4,10.4_ = 7.322; *P* = 0.014; *n* = 8) (Figure 5C).

### Trigeminovascular processing through VTA^PBP^ modulation results in reduced blood glucose levels

In all experiments, blood glucose was within physiological levels before microinjection of drugs (5.47 ± 0.09 mmol/L). Data from 48 experiments were analyzed, and in animals that received two microinjections, blood glucose measures were only taken after the first microinjection. Modulation with glutamate significantly decreased blood glucose levels by 11% (*t*_7_ = 2.981; *P* = 0.020; *n* = 8), an effect mimicked by bicuculline (*t*_9_ = 3.641; *P* = 0.005; *n* = 10). Furthermore, naratriptan VTA^PBP^ modulation significantly decreased blood glucose levels by 12% (*t*_7_ = 2.443; *P* = 0.045; *n* = 8) and PACAP38 induced a significant blood glucose level reduction of 17% (*t*_6_ = 2.731; *P* = 0.034; *n* = 7). There were no significant effects on blood glucose levels following microinjection of quinpirole (*t*_7_ = 0.082; *P* = 0.937; *n* = 8) or vehicle control (*t*_6_ = 1.485; *P* = 0.188; *n* = 7). Blood glucose levels before and 60 min following VTA^PBP^ microinjection are presented in Table S2.

## Discussion

We show that pharmacological modulation of the VTA^PBP^ is sufficient to alter the transmission of trigeminovascular nociceptive and innocuous inputs. Whether VTA^PBP^ activity is necessary for physiological trigeminovascular sensory processing is unknown, yet our findings and others showing VTA activation in human imaging of migraine (3) and VTA involvement in aversive behavior in an inflammatory model of headache (8) suggest that the VTA may be relevant to understand associated sensory and homeostatic symptoms in susceptible migraine patients. Anatomical studies (9,52) do not report direct VTA projections to the TCC, therefore the effects of VTA^PBP^ manipulation on TCC neuronal responses can be explained by indirect action on relay stations, namely the NAc, PAG, PVN or lateral hypothalamus (LH) (45,53).

We showed that excitatory and inhibitory inputs to the VTA^PBP^ inhibit trigeminovascular neuronal responses. Within the VTA, there are two general populations of GABA neurons: interneurons, which provide local inhibition of DA neurons, and projection neurons, which provide long-range inhibition of multiple brain areas including the NAc, the prefrontal cortex, the lateral habenula, lateral hypothalamus, preoptic area, and amygdala, as well as to structures in the thalamus, midbrain, pons, and medulla (14,54,55). Importantly, these VTA GABA neurons are known to express the GABA_A_ receptor (55–57). Given the current literature (6,11,57–59), possible explanations could account for the effects of the GABA_A_ receptor antagonist (bicuculline): 1) Effects directly on VTA^PBP^ Daergic neurons expressing GABA_A_ receptors, preventing GABAergic inhibition, and thereby increasing Daergic firing (thus inducing a similar response to glutamate VTA^PBP^ microinjection); 2) Effects on VTA GABA interneurons, facilitating its firing and thereby increasing inhibitory input to VTA^PBP^ DA neurons, resulting in inhibition of the mesolimbic DA system; 3) Effects on GABAergic VTA^PBP^ long-range projection neurons, facilitating its firing, leading to increased inhibition of target structures (e.g. NAc, prefrontal cortex, central amygdala, dorsal raphe nucleus, among other regions). Although many of these projections have been identified anatomically, there has been little functional characterization of cellular properties of these neurons or synaptic properties of their terminals and projection neurons may have local collaterals, allowing coordination of VTA activity with activity in distal target regions (60). Overall, given that DA neurons represent 65% of and GABA neurons 30% of VTA neurons (11), one can hypothesize that the effects observed herein are likely to be mediated through VTA^PBP^ Daergic neurons expressing GABA_A_ receptors.

Of note, it has been difficult to disentangle the function of DA neurons and GABA neurons, and between local VTA GABA neurons and GABAergic projection neurons (extensively reviewed elsewhere (59,60)). In addition, VTA neurons release various combinations of DA, GABA, and glutamate, all of which form local and long-range connections (9,15,25,52,61). Therefore, to gain a full understanding of the likely neuronal circuitry underlying the responses herein, it is imperative to gain genetic access to these cells (e.g. opto- or chemogenetics) in future studies of migraine pathophysiology.

### Naratriptan and PACAP38 in the VTAPBP exert similar effects in TCC neuronal firing

Naratriptan in the VTA^PBP^ inhibits TCC neuronal firing through 5-HT_1B/1D_ receptors, putatively present throughout this structure. Consistent with our findings, naratriptan has similar effects when microinjected into the vlPAG (47), PVN (45), A11 (34) and infused peripherally (44). Given that serotonin is implicated in reward processing (62) and naratriptan-induced 5-HT_1B/1D_ receptor activation is able to block nociceptive pathways, our findings support the rewarding effect of pain relief.

Unexpectedly, PACAP38 in the VTA^PBP^ reduced all types of TCC activities. These results are challenging given that PACAP38 administered intravenously induces delayed sensitization of central trigeminovascular neurons (50), which translates to delayed migraine-like headaches in 50% of migraine patients without aura (33). In animals, PACAP38 in the PVN does not modify Aδ-fiber responses but increases TCC spontaneous activity (45). Nonetheless, PACAP’s role in pain transmission is complex, with studies showing both anti- and pro-nociceptive actions (63,64). In our study, the precise mechanisms through which intra-VTA^PBP^ PACAP38 inhibits TCC responses are unknown, although the differences on nociceptive behavior might be receptor dependent.

The immunohistochemistry in our study showed the putative expression of three PACAP receptors specifically within the VTA^PBP^. Previously, *in situ* hybridization revealed a wide distribution of PACAP-R mRNA with intense-to-moderate labeling in the VTA (30), yet an immunoreactive study showed no expression above background density level for PAC_1_, <40% of maximal level for VPAC_1_, and <80% of maximal level for VPAC_2_ (32).

Regarding peptide brain expression, soma that contain PACAP are relatively restricted to selected regions of the limbic system and brainstem, yet fibers containing PACAP and levels of PACAP protein are found in the VTA (65). To date, it is known that major PACAP inputs to the VTA originate from the hypothalamic ventromedial nucleus (VMN) and these neurons inhibit the excitability of VTA dopaminergic neurons *via* activation of PAC_1_ receptors and K_ATP_ channels (66). Conversely, vasoactive intestinal peptide (VIP), which shows 68% sequence homology with PACAP and acts on the same receptors (although PAC_1_ shows much greater affinity for PACAP than for VIP) (67), is highly expressed in the VTA (68–70) and is co-expressed with TH in the VTA^PBP^ (68).

While we confirm PAC_1,_ VPAC_1_ and VPAC_2_ receptors are “putatively” present specifically within the VTA^PBP^, it has been suggested PACAP pro-nociceptive actions are likely mediated *via* PAC_1_ receptor (71) and anti-nociceptive, anti-hyperalgesic and anti-allodynic effects mediated by VPAC_1_ and VPAC_2_ receptors (72). Therefore, additional studies (e.g. knock-out receptor) are needed to clarify this mechanism.

Moreover, recent studies provide evidence that PACAP plays an important role in reward processing, such as modulation of the rewarding and reinforcing actions of addictive drugs (65). PACAP in specific limbic brain regions can promote reward seeking and intake and itself is stimulated by their intake (65). Although the expression and wide distribution of PAC_1_ and/or VPAC receptors contribute to the broad and diverse actions of PACAP, the stress- and addiction-related responses of PACAP appear to be mediated predominantly by the PACAP-selective PAC_1_ receptor (73). Of note, there is a close relationship between reward/aversion and pain relief/pain mediated by the mesolimbic reward circuitry that involves the mesolimbic DA emanating from the VTA and projecting to the NAc (74,75). It has been also postulated that removal of an aversive state is rewarding (76). Indeed, relief of pain produces negative reinforcement through activation of the mesolimbic reward–valuation circuitry. In specific, activation of VTA dopaminergic neurons and release of DA as well as activation of dopaminergic receptors in the NAc mediates the reinforcing effect of pain relief (74,77,78). Given the role of PACAP on motivated behaviors in response to addictive drugs and that addiction behavior is associated with reward anticipation (e.g. craving), it is possible that PACAP, predominantly *via* the PAC_1_ receptor, may likely contribute to a mechanism of reward processing and craving involving inhibition of nociceptive responses as a way of termination of the aversive stimulus. However, we cannot exclude the fact that PACAP binds with high affinity to PAC_1_ and VPAC_1_ and VPAC_2_, and given this overlap, it will be important to dissect which receptor is mediating a given PACAP effect.

Regarding DA signaling, quinpirole administration in the VTA^PBP^ inhibited TCC neuronal firing; an effect supported by systemic studies (34). Dopamine D_2_-like receptors are important in pain modulation (79) and exert auto-inhibitory somatodendritic effects (80). Thus, if quinpirole is likely to induce auto-inhibitory actions on DA neurons and, herein, inhibited TCC noxious inputs, it is possible VTA^PBP^ non-DA neurons (e.g. GABAergic neurons) could have influenced TCC nociceptive processing in our experiments.

Overall, clinical-relevant drugs in the VTA^PBP^ modulate both spontaneous and dural-evoked activities of TCC neurons suggesting its effects are not nociceptive-specific. Given that modulation of VTA^PBP^ neuronal activity is sufficient to elicit changes in trigeminovascular sensory processing, it is possible that, in susceptible persons, VTA^PBP^ neurons could alter trigeminovascular processing by integrating nociceptive, as well as other processing mechanisms (e.g. hedonic or homeostatic). In addition, the modulatory influences of VTA^PBP^ on both spontaneous and dural-evoked TCC activities could be mediated by different top-down mechanisms promoted by the varied neuronal populations within the VTA^PBP^.

The VTA is known to process mechanical inputs through DAergic mechanisms (81). We show that VTA^PBP^ modulation by naratriptan and PACAP38 alters responses to facial mechanical stimulation, which may be relevant for underlying allodynia and hyperalgesia mechanisms in susceptible patients. Interestingly, a previous study has demonstrated a relationship between DA and 5-HT within the TCC *via* DA-projecting A11 neurons, where lesioning the A11 facilitates noxious facial-evoked inputs, and both systemic 5-HT_1B/1D_ and D_2_ receptor agonists reverse this (34). Thus, it is possible that a similar synergistic relationship occurs as well in the TCC *via* VTA^PBP^.

### Modulation of the VTA^PBP^ impacts post-trigeminovascular processing glycemia

Trigeminovascular sensory processing through VTA^PBP^ modulation is accompanied by reduced blood glucose levels. We have previously shown that dural-evoked TCC neuronal firing is modulated by systemic glucoregulatory peptides and TCC pERK1/2 cell expression is decreased by insulin and increased by glucagon (35). Moreover, disturbances in glucose and insulin metabolism have been reported in migraine patients, namely impaired insulin sensitivity (extensively reviewed elsewhere (28)).

These electrophysiological studies were carried out in anesthetized animals, yet we confirmed a normoglycemic state within physiological levels, ruling out possible anesthetic-induced variations.

We hypothesize that the reduced blood glucose levels may occur due to specific metabolic effects triggered by VTA^PBP^ modulation and its outcomes on projection targets. Possible metabolic effects include increased insulin secretion, increased expression of insulin sensitive glucose transporters (GLUTs), increased insulin-mediated suppression of endogenous glucose production, and improved insulin sensitivity with stimulation of glucose utilization in peripheral tissues. To our knowledge, none of these have been studied as an effect of VTA neuronal activity. Although the VTA neuronal firing is influenced by glucoregulatory peptides, including insulin (82,83) and glucagon-like peptide-1 (GLP-1) (84,85), there is no evidence reporting VTA neuronal activity direct effects on systemic glucose levels. More recently, however, it has been shown that NAc neuronal activity (a known VTA projection target) seems to regulate systemic glucose metabolism by increasing insulin sensitivity (86). Given the current available literature, we may speculate the NAc could potentially be a relay station explaining the reduced glucose levels observed in our study.

Moreover, naratriptan and PACAP are known to influence glycemia. Serotonin interacts with glucose metabolism (87) and induces a dose-dependent serum insulin increase and serum glucose decrease in mice (88,89); whereas in obese diabetic animals and diabetic humans serotonergic drugs improve glucose tolerance and insulin sensitivity (90–92). PACAP is synthesized and released by pancreatic *β*-cells and exerts several metabolic actions, such as stimulation of insulin production and release, and increase of insulin-induced glucose uptake in adipocytes, thereby improving glucose tolerance (93–95). It is possible that improved insulin sensitivity may accompany naratriptan and PACAP-38 antinociceptive effects in our study. Whether there is a nociceptive-specific glucoregulatory mechanism in migraine remains to be established.

Physiologically, blood glucose changes may lead to appetite changes. In susceptible migraine patients, the interplay between VTA^PBP^ activity and the sensory processing by the trigeminovascular system may be relevant to understand associated sensory and homeostatic symptoms. Our findings may provide plausible groundwork biology to unravel appetite changes in susceptible migraine patients.

### Further research and implications

Given that VTA is a heterogeneous nucleus containing dopaminergic (DA) neurons (9), as well as GABAergic and GLU neurons (10–12), additional experiments could dissect the co-localization of 5-HT_1_ and PACAP receptors with either TH, Glu or GABAergic neurons, providing more information on the likely neuronal circuitry mediating PACAP and naratriptan effects on VTA^PBP^ neurons.

Furthermore, a technical constraint of the study is that the methodology used herein does not unravel the likely neuronal circuitry mediating PACAP and naratriptan effects on VTA^PBP^ neurons. For instance, brain slice recordings could provide additional information, such as identification of the VTA^PBP^ neuronal type being modulated by these drugs. In addition, a direct input from the VTA to the TCC is currently unknown. Regardless, it has been shown in an animal model of orofacial pain, that stimulation of the NAc inhibited nociceptive trigeminal nucleus caudalis neurons mediated by NAc dopamine D2 receptors and transmitted through the rostral ventromedial medulla (96). Given the known VTA^PBP^-NAc projections, future experiments could incorporate pathway-specific manipulation of this brain circuitry in a migraine animal model by using opto- or chemogenetics.

## Article highlights

- Modulation of VTA^PBP^ neuronal activity is sufficient to alter the transmission of trigeminovascular nociceptive and innocuous inputs.
- Serotonin and PACAP receptors are putatively expressed within the VTA^PBP^ region.
- Naratriptan, PACAP38, quinpirole intra-VTA^PBP^ inhibit trigeminovascular responses.
- Trigeminovascular sensory processing through VTA^PBP^ modulation is accompanied by reduced blood glucose levels.

## Supplemental Material

sj-pdf-1-cep-10.1177_03331024221110111 - Supplemental material for Pharmacological modulation of ventral tegmental area neurons elicits changes in trigeminovascular sensory processing and is accompanied by glycemic changes: Implications for migraine Click here for additional data file. Supplemental material, sj-pdf-1-cep-10.1177_03331024221110111 for Pharmacological modulation of ventral tegmental area neurons elicits changes in trigeminovascular sensory processing and is accompanied by glycemic changes: Implications for migraine by Margarida Martins-Oliveira, Simon Akerman, Philip R Holland, Isaura Tavares and Peter J Goadsby in Cephalalgia

## CRediT Author Statement

**Margarida Martins-Oliveira:** Conceptualization, Methodology, Formal analysis, Investigation, Writing – Original Draft, Writing – Review & Editing, Visualization, Funding acquisition.

**Simon Akerman:** Conceptualization, Methodology, Supervision, Writing – Review & Editing, Project administration.

**Philip R Holland:** Conceptualization, Methodology, Supervision, Writing – Review & Editing, Project administration, Funding acquisition.

**Isaura Tavares:** Conceptualization, Methodology, Supervision, Writing – Review & Editing, Project administration, Funding acquisition.

**Peter J Goadsby:** Conceptualization, Methodology, Supervision, Writing – Review & Editing, Project administration, Funding acquisition.

## References

[1] Denuelle M Fabre N Payoux P, et al. Hypothalamic activation in spontaneous migraine attacks. Headache 2007; 47: 1418–1426. 18052951 10.1111/j.1526-4610.2007.00776.x

[2] Schulte LH May A. The migraine generator revisited: continuous scanning of the migraine cycle over 30 days and three spontaneous attacks. Brain 2016; 139: 1987–1993. 27190019 10.1093/brain/aww097

[3] Maniyar FH Sprenger T Monteith T, et al. Brain activations in the premonitory phase of nitroglycerin-triggered migraine attacks. Brain 2014; 137: 232–241. 10.1093/brain/awt320 24277718

[4] Mercer Lindsay N Chen C Gilam G, et al. Brain circuits for pain and its treatment. Sci Transl Med 2021; 13: eabj7360. 34757810 10.1126/scitranslmed.abj7360 PMC8675872

[5] Ferrario CR Labouèbe G Liu S, et al. homeostasis meets motivation in the battle to control food intake. J Neurosci 2016; 36: 11469–11481. 27911750 10.1523/JNEUROSCI.2338-16.2016 PMC5125214

[6] Morales M Margolis EB. Ventral tegmental area: cellular heterogeneity, connectivity and behaviour. Nat Rev Neurosci 2017; 18: 73–85. 28053327 10.1038/nrn.2016.165

[7] Hsu TM McCutcheon JE Roitman MF. Parallels and overlap: the integration of homeostatic signals by mesolimbic dopamine neurons. Front Psychiatry 2018; 9: 410. 30233430 10.3389/fpsyt.2018.00410 PMC6129766

[8] Waung MW Margolis EB Charbit AR, et al. A midbrain circuit that mediates headache aversiveness in rats. Cell Rep 2019; 28: 2739–2747.e2734. 31509737 10.1016/j.celrep.2019.08.009 PMC6831085

[9] Swanson LW. The projections of the ventral tegmental area and adjacent regions: a combined fluorescent retrograde tracer and immunofluorescence study in the rat. Brain Res Bull 1982; 9: 321–353. 6816390 10.1016/0361-9230(82)90145-9

[10] Dobi A Margolis EB Wang HL, et al. Glutamatergic and nonglutamatergic neurons of the ventral tegmental area establish local synaptic contacts with dopaminergic and nondopaminergic neurons. J Neurosci 2010; 30: 218–229. 20053904 10.1523/JNEUROSCI.3884-09.2010 PMC3209506

[11] Margolis EB Toy B Himmels P, et al. Identification of rat ventral tegmental area GABAergic neurons. PLoS One 2012; 7: e42365. 22860119 10.1371/journal.pone.0042365 PMC3409171

[12] Yamaguchi T Sheen W Morales M. Glutamatergic neurons are present in the rat ventral tegmental area. Eur J Neurosci 2007; 25: 106–118. 17241272 10.1111/j.1460-9568.2006.05263.x PMC3209508

[13] Nair-Roberts RG Chatelain-Badie SD Benson E, et al. Stereological estimates of dopaminergic, GABAergic and glutamatergic neurons in the ventral tegmental area, substantia nigra and retrorubral field in the rat. Neuroscience 2008; 152: 1024–1031. 18355970 10.1016/j.neuroscience.2008.01.046 PMC2575227

[14] Taylor SR Badurek S Dileone RJ, et al. GABAergic and glutamatergic efferents of the mouse ventral tegmental area. J Comp Neurol 2014; 522: 3308–3334. 24715505 10.1002/cne.23603 PMC4107038

[15] Morales M Root DH. Glutamate neurons within the midbrain dopamine regions. Neuroscience 2014; 282C: 60–68. 10.1016/j.neuroscience.2014.05.032 PMC4397110 24875175

[16] Fields HL Hjelmstad GO Margolis EB, et al. Ventral tegmental area neurons in learned appetitive behavior and positive reinforcement. Annu Rev Neurosci 2007; 30: 289–316. 17376009 10.1146/annurev.neuro.30.051606.094341

[17] Gorelova N Mulholland PJ Chandler LJ, et al. The glutamatergic component of the mesocortical pathway emanating from different subregions of the ventral midbrain. Cereb Cortex 2012; 22: 327–336. 21666135 10.1093/cercor/bhr107 PMC3256405

[18] Yamaguchi T Wang HL Li X, et al. Mesocorticolimbic glutamatergic pathway. J Neurosci 2011; 31: 8476–8490. 21653852 10.1523/JNEUROSCI.1598-11.2011 PMC6623324

[19] Hnasko TS Hjelmstad GO Fields HL, et al. Ventral tegmental area glutamate neurons: electrophysiological properties and projections. J Neurosci 2012; 32: 15076–15085. 23100428 10.1523/JNEUROSCI.3128-12.2012 PMC3685320

[20] Sanchez-Catalan MJ Kaufling J Georges F, et al. The antero-posterior heterogeneity of the ventral tegmental area. Neuroscience 2014; 282C: 198–216. 10.1016/j.neuroscience.2014.09.025 25241061

[21] Oades RD Halliday GM. Ventral tegmental (A10) system: neurobiology. 1. Anatomy and connectivity. Brain Res 1987; 434: 117–165. 3107759 10.1016/0165-0173(87)90011-7

[22] Paxinos G Watson C. The Rat Brain in Stereotaxic Coordinates. 5th ed. San Diego, California: Elsevier Academic Press, 2005.

[23] Mazei-Robison MS Nestler EJ. Opiate-induced molecular and cellular plasticity of ventral tegmental area and locus coeruleus catecholamine neurons. Cold Spring Harb Perspect Med 2012; 2: a012070. 22762025 10.1101/cshperspect.a012070 PMC3385942

[24] Lammel S Hetzel A Häckel O, et al. Unique properties of mesoprefrontal neurons within a dual mesocorticolimbic dopamine system. Neuron 2008; 57: 760–773. 18341995 10.1016/j.neuron.2008.01.022

[25] Lammel S Ion DI Roeper J, et al. Projection-specific modulation of dopamine neuron synapses by aversive and rewarding stimuli. Neuron 2011; 70: 855–862. 21658580 10.1016/j.neuron.2011.03.025 PMC3112473

[26] Yang H de Jong JW Tak Y, et al. Nucleus accumbens subnuclei regulate motivated behavior via direct inhibition and disinhibition of VTA dopamine subpopulations. Neuron 2018; 97: 434–449.e434. 29307710 10.1016/j.neuron.2017.12.022 PMC5773387

[27] Giffin NJ Ruggiero L Lipton RB, et al. Premonitory symptoms in migraine: an electronic diary study. Neurology 2003; 60: 935–940. 12654956 10.1212/01.wnl.0000052998.58526.a9

[28] Martins-Oliveira M Tavares I Goadsby PJ. Was it something I ate? Understanding the bidirectional interaction of migraine and appetite neural circuits. Brain Res 2021: 147629. DOI: 10.1016/j.brainres.2021.147629. 10.1016/j.brainres.2021.147629 34428465

[29] Goadsby PJ Holland PR Martins-Oliveira M, et al. Pathophysiology of migraine: A disorder of sensory processing. Physiol Rev 2017; 97: 553–622. 28179394 10.1152/physrev.00034.2015 PMC5539409

[30] Hashimoto H Nogi H Mori K, et al. Distribution of the mRNA for a pituitary adenylate cyclase-activating polypeptide receptor in the rat brain: an in situ hybridization study. J Comp Neurol 1996; 371: 567–577. 8841910 10.1002/(SICI)1096-9861(19960805)371:4<567::AID-CNE6>3.0.CO;2-2

[31] Pazos A Palacios JM. Quantitative autoradiographic mapping of serotonin receptors in the rat brain. I. Serotonin-1 receptors. Brain Res 1985; 346: 205–230. 4052776 10.1016/0006-8993(85)90856-x

[32] Joo KM Chung YH Kim MK, et al. Distribution of vasoactive intestinal peptide and pituitary adenylate cyclase-activating polypeptide receptors (VPAC1, VPAC2, and PAC1 receptor) in the rat brain. J Comp Neurol 2004; 476: 388–413. 15282712 10.1002/cne.20231

[33] Schytz HW Birk S Wienecke T, et al. PACAP38 induces migraine-like attacks in patients with migraine without aura. Brain 2009; 132: 16–25. 19052139 10.1093/brain/awn307

[34] Charbit AR Akerman S Goadsby PJ. Trigeminocervical complex responses after lesioning dopaminergic A11 nucleus are modified by dopamine and serotonin mechanisms. Pain 2011; 152: 2365–2376. 21868165 10.1016/j.pain.2011.07.002

[35] Martins-Oliveira M Akerman S Holland PR, et al. Neuroendocrine signaling modulates specific neural networks relevant to migraine. Neurobiol Dis 2017; 101: 16–26. 28108291 10.1016/j.nbd.2017.01.005 PMC5356993

[36] Martins-Oliveira M Akerman S Holland P, et al. Midbrain reward pathway and premonitory food craving in migraineurs: studies in a animal model. Cephalalgia 2016; 36: 1–185.

[37] Martins-Oliveira M Akerman S Holland PR, et al. Pleasure and pain: exploring neurobiological mechanisms of food craving before migraine pain. Cephalalgia 2017; 37: 1–378.28880583

[38] Kilkenny C Browne W Cuthill IC, et al. Animal research: reporting in vivo experiments: the ARRIVE guidelines. Br J Pharmacol 2010; 160: 1577–1579. 20649561 10.1111/j.1476-5381.2010.00872.x PMC2936830

[39] Zimmermann M. Ethical guidelines for investigations of experimental pain in conscious animals. Pain 1983; 16: 109–110. 6877845 10.1016/0304-3959(83)90201-4

[40] Pommer S Akamine Y Schiffmann SN, et al. The effect of serotonin receptor 5-HT1B on lateral inhibition between spiny projection neurons in the mouse striatum. J Neurosci 2021; 41: 7831–7847. 10.1523/JNEUROSCI.1037-20.2021 PMC8445048 34348999

[41] Varodayan FP Minnig MA Steinman MQ, et al. PACAP regulation of central amygdala GABAergic synapses is altered by restraint stress. Neuropharmacol 2020; 168: 107752. 10.1016/j.neuropharm.2019.107752 PMC7048635 31476352

[42] Minnig MA Park T Echeveste Sanchez M, et al. Viral-mediated knockdown of nucleus accumbens shell pac1 receptor promotes excessive alcohol drinking in alcohol-preferring rats. Front Behav Neurosci 2021; 15: 787362. 34924973 10.3389/fnbeh.2021.787362 PMC8678417

[43] Ivic I Balasko M Fulop BD, et al. VPAC1 receptors play a dominant role in PACAP-induced vasorelaxation in female mice. PLoS One 2019; 14: e0211433. 30682157 10.1371/journal.pone.0211433 PMC6347420

[44] Martins-Oliveira M Akerman S Tavares I, et al. Neuropeptide Y inhibits the trigeminovascular pathway through NPY Y1 receptor: implications for migraine. Pain 2016; 157: 1666–1673. 27023421 10.1097/j.pain.0000000000000571 PMC4949002

[45] Robert C Bourgeais L Arreto CD, et al. Paraventricular hypothalamic regulation of trigeminovascular mechanisms involved in headaches. J Neurosci 2013; 33: 8827–8840. 23678125 10.1523/JNEUROSCI.0439-13.2013 PMC6618837

[46] Knight YE Bartsch T Goadsby PJ. Trigeminal antinociception induced by bicuculline in the periaqueductal gray (PAG) is not affected by PAG P/Q-type calcium channel blockade in rat. Neurosci Lett 2003; 336: 113–116. 12499053 10.1016/s0304-3940(02)01250-8

[47] Bartsch T Knight YE Goadsby PJ. Activation of 5-HT(1B/1D) receptor in the periaqueductal gray inhibits nociception. Ann Neurol 2004; 56: 371–381. 15349864 10.1002/ana.20193

[48] Zhang DG Amin FM Guo S, et al. Plasma glucose levels increase during spontaneous attacks of migraine with and without aura. Headache 2020; 60: 655–664. 32031249 10.1111/head.13760

[49] Rainero I Limone P Ferrero M, et al. Insulin sensitivity is impaired in patients with migraine. Cephalalgia 2005; 25: 593–597. 16033384 10.1111/j.1468-2982.2005.00928.x

[50] Akerman S Goadsby PJ. Neuronal PAC1 receptors mediate delayed activation and sensitization of trigeminocervical neurons: Relevance to migraine. Sci Transl Med 2015; 7: 308ra157. 10.1126/scitranslmed.aaa7557 26446954

[51] Field A. Discovering Statistics Using SPSS. 4 ed. London: SAGE, 2013.

[52] Aransay A Rodríguez-López C García-Amado M, et al. Long-range projection neurons of the mouse ventral tegmental area: a single-cell axon tracing analysis. Front Neuroanat 2015; 9: 59. 26042000 10.3389/fnana.2015.00059 PMC4436899

[53] Becerra L Navratilova E Porreca F, et al. Analogous responses in the nucleus accumbens and cingulate cortex to pain onset (aversion) and offset (relief) in rats and humans. J Neurophysiol 2013; 110: 1221–1226. 23785130 10.1152/jn.00284.2013 PMC3763092

[54] van Zessen R Phillips JL Budygin EA, et al. Activation of VTA GABA neurons disrupts reward consumption. Neuron 2012; 73: 1184–1194. 22445345 10.1016/j.neuron.2012.02.016 PMC3314244

[55] Tan KR Yvon C Turiault M, et al. GABA neurons of the VTA drive conditioned place aversion. Neuron 2012; 73: 1173–1183. 22445344 10.1016/j.neuron.2012.02.015 PMC6690362

[56] Tan KR Brown M Labouèbe G, et al. Neural bases for addictive properties of benzodiazepines. Nature 2010; 463: 769–774. 20148031 10.1038/nature08758 PMC2871668

[57] Ciccarelli A Calza A Panzanelli P, et al. Organization of GABAergic synaptic circuits in the rat ventral tegmental area. PLoS One 2012; 7: e46250. 23056271 10.1371/journal.pone.0046250 PMC3466259

[58] Ikemoto S Kohl RR McBride WJ. GABA(A) receptor blockade in the anterior ventral tegmental area increases extracellular levels of dopamine in the nucleus accumbens of rats. J Neurochem 1997; 69: 137–143. 9202304 10.1046/j.1471-4159.1997.69010137.x

[59] Creed MC Ntamati NR Tan KR. VTA GABA neurons modulate specific learning behaviors through the control of dopamine and cholinergic systems. Front Behav Neurosci 2014; 8: 8. 20140122. 10.3389/fnbeh.2014.00008 PMC3897868 24478655

[60] Bouarab C Thompson B Polter AM. VTA GABA neurons at the interface of stress and reward. Front Neural Circuits 2019; 13: 78. 31866835 10.3389/fncir.2019.00078 PMC6906177

[61] Lammel S Lim BK Ran C, et al. Input-specific control of reward and aversion in the ventral tegmental area. Nature 2012; 491: 212–217. 23064228 10.1038/nature11527 PMC3493743

[62] Kranz GS Kasper S Lanzenberger R. Reward and the serotonergic system. Neuroscience 2010; 166: 1023–1035. 20109531 10.1016/j.neuroscience.2010.01.036

[63] Sándor K Kormos V Botz B, et al. Impaired nocifensive behaviours and mechanical hyperalgesia, but enhanced thermal allodynia in pituitary adenylate cyclase-activating polypeptide deficient mice. Neuropeptides 2010; 44: 363–371. 20621353 10.1016/j.npep.2010.06.004

[64] Shimizu T Katahira M Sugawara H, et al. Diverse effects of intrathecal pituitary adenylate cyclase-activating polypeptide on nociceptive transmission in mice spinal cord. Regul Pept 2004; 123: 117–122. 15518901 10.1016/j.regpep.2004.05.019

[65] Gargiulo AT Curtis GR Barson JR. Pleiotropic pituitary adenylate cyclase-activating polypeptide (PACAP): Novel insights into the role of PACAP in eating and drug intake. Brain Res 2020; 1729: 146626. 31883848 10.1016/j.brainres.2019.146626 PMC6953419

[66] Le N Hernandez J Gastelum C, et al. Pituitary adenylate cyclase activating polypeptide inhibits A10 dopamine neurons and suppresses the binge-like consumption of palatable food. Neuroscience 2021; 478: 49–64. 34597709 10.1016/j.neuroscience.2021.09.016 PMC8608708

[67] Harmar AJ Fahrenkrug J Gozes I, et al. Pharmacology and functions of receptors for vasoactive intestinal peptide and pituitary adenylate cyclase-activating polypeptide: IUPHAR review 1. Br J Pharmacol 2012; 166: 4–17. 22289055 10.1111/j.1476-5381.2012.01871.x PMC3415633

[68] Tiklová K Björklund Å Lahti L, et al. Single-cell RNA sequencing reveals midbrain dopamine neuron diversity emerging during mouse brain development. Nat Commun 2019; 10: 581. 30718509 10.1038/s41467-019-08453-1 PMC6362095

[69] Poulin JF Gaertner Z Moreno-Ramos OA, et al. Classification of midbrain dopamine neurons using single-cell gene expression profiling approaches. Trends Neurosci 2020; 43: 155–169. 32101709 10.1016/j.tins.2020.01.004 PMC7285906

[70] Chung CY Seo H Sonntag KC, et al. Cell type-specific gene expression of midbrain dopaminergic neurons reveals molecules involved in their vulnerability and protection. Hum Mol Genet 2005; 14: 1709–1725. 15888489 10.1093/hmg/ddi178 PMC2674782

[71] Yokai M Kurihara T Miyata A. Spinal astrocytic activation contributes to both induction and maintenance of pituitary adenylate cyclase-activating polypeptide type 1 receptor-induced long-lasting mechanical allodynia in mice. Mol Pain 2016; 12. DOI: 10.1177/1744806916646383. 10.1177/1744806916646383 PMC4956379 27175011

[72] Sándor K Bölcskei K McDougall JJ, et al. Divergent peripheral effects of pituitary adenylate cyclase-activating polypeptide-38 on nociception in rats and mice. Pain 2009; 141: 143–150. 19091468 10.1016/j.pain.2008.10.028

[73] Miles OW May V Hammack SE. Pituitary Adenylate Cyclase-Activating Peptide (PACAP) signaling and the dark side of addiction. J Mol Neurosci 2019; 68: 453–464. 30074172 10.1007/s12031-018-1147-6 PMC6732790

[74] Navratilova E Porreca F. Reward and motivation in pain and pain relief. Nat Neurosci 2014; 17: 1304–1312. 25254980 10.1038/nn.3811 PMC4301417

[75] Lammel S Lim BK Malenka RC. Reward and aversion in a heterogeneous midbrain dopamine system. Neuropharmacology 2014; 76 Pt B: 351–359. 23578393 10.1016/j.neuropharm.2013.03.019 PMC3778102

[76] Tanimoto H Heisenberg M Gerber B. Experimental psychology: event timing turns punishment to reward. Nature 2004; 430: 983. 15329711 10.1038/430983a

[77] De Felice M Eyde N Dodick D, et al. Capturing the aversive state of cephalic pain preclinically. Ann Neurol 2013; 74: 257–265. 23686557 10.1002/ana.23922 PMC3830648

[78] Navratilova E Xie JY Okun A, et al. Pain relief produces negative reinforcement through activation of mesolimbic reward-valuation circuitry. Proc Natl Acad Sci U S A 2012; 109: 20709–20713. 23184995 10.1073/pnas.1214605109 PMC3528534

[79] Moradi M Yazdanian M Haghparast A. Role of dopamine D2-like receptors within the ventral tegmental area and nucleus accumbens in antinociception induced by lateral hypothalamus stimulation. Behav Brain Res 2015; 292: 508–514. 26166189 10.1016/j.bbr.2015.07.007

[80] Beckstead MJ Grandy DK Wickman K, et al. Vesicular dopamine release elicits an inhibitory postsynaptic current in midbrain dopamine neurons. Neuron 2004; 42: 939–946. 15207238 10.1016/j.neuron.2004.05.019

[81] Sogabe S Yagasaki Y Onozawa K, et al. Mesocortical dopamine system modulates mechanical nociceptive responses recorded in the rat prefrontal cortex. BMC Neurosci 2013; 14: 65. 23815681 10.1186/1471-2202-14-65 PMC3710228

[82] Liu S Borgland SL. Regulation of the mesolimbic dopamine circuit by feeding peptides. Neuroscience 2015; 289: 19–42. 25583635 10.1016/j.neuroscience.2014.12.046

[83] Mebel DM Wong JC Dong YJ, et al. Insulin in the ventral tegmental area reduces hedonic feeding and suppresses dopamine concentration via increased reuptake. Eur J Neurosci 2012; 36: 2336–2346. 22712725 10.1111/j.1460-9568.2012.08168.x PMC5239666

[84] Konanur VR Hsu TM Kanoski SE, et al. Phasic dopamine responses to a food-predictive cue are suppressed by the glucagon-like peptide-1 receptor agonist Exendin-4. Physiol Behav 2020; 215: 112771. 31821815 10.1016/j.physbeh.2019.112771 PMC8112128

[85] Dickson SL Shirazi RH Hansson C, et al. The glucagon-like peptide 1 (GLP-1) analogue, exendin-4, decreases the rewarding value of food: a new role for mesolimbic GLP-1 receptors. J Neurosci 2012; 32: 4812–4820. 22492036 10.1523/JNEUROSCI.6326-11.2012 PMC6620919

[86] Ter Horst KW Lammers NM Trinko R, et al. Striatal dopamine regulates systemic glucose metabolism in humans and mice. Sci Transl Med 2018; 10. DOI: 10.1126/scitranslmed.aar3752. 10.1126/scitranslmed.aar3752 29794060

[87] McGlashon JM Gorecki MC Kozlowski AE, et al. Central serotonergic neurons activate and recruit thermogenic brown and beige fat and regulate glucose and lipid homeostasis. Cell Metab 2015; 21: 692–705. 25955206 10.1016/j.cmet.2015.04.008 PMC4565052

[88] Yamada J Sugimoto Y Kimura I, et al. Serotonin-induced hypoglycemia and increased serum insulin levels in mice. Life Sci 1989; 45: 1931–1936. 2689822 10.1016/0024-3205(89)90547-x

[89] Sugimoto Y Kimura I Yamada J, et al. Effects of serotonin on blood glucose and insulin levels of glucose- and streptozotocin-treated mice. Jpn J Pharmacol 1990; 54: 93–96. 2148781 10.1254/jjp.54.93

[90] Arora R Dryden S McKibbin PE, et al. Acute dexfenfluramine administration normalizes glucose tolerance in rats with insulin-deficient diabetes. Eur J Clin Invest 1994; 24: 182–187. 8033952 10.1111/j.1365-2362.1994.tb00986.x

[91] Gomez R Huber J Tombini G, et al. Acute effect of different antidepressants on glycemia in diabetic and non-diabetic rats. Braz J Med Biol Res 2001; 34: 57–64. 11151029 10.1590/s0100-879x2001000100007

[92] Maheux P Ducros F Bourque J, et al. Fluoxetine improves insulin sensitivity in obese patients with non-insulin-dependent diabetes mellitus independently of weight loss. Int J Obes Relat Metab Disord 1997; 21: 97–102. 9043962 10.1038/sj.ijo.0800372

[93] Yada T Sakurada M Ihida K, et al. Pituitary adenylate cyclase activating polypeptide is an extraordinarily potent intra-pancreatic regulator of insulin secretion from islet beta-cells. J Biol Chem 1994; 269: 1290–1293. 8288592

[94] Nakata M Shioda S Oka Y, et al. Insulinotropin PACAP potentiates insulin-stimulated glucose uptake in 3T3 L1 cells. Peptides 1999; 20: 943–948. 10503772 10.1016/s0196-9781(99)00085-6

[95] Nakata M Yada T. PACAP in the glucose and energy homeostasis: physiological role and therapeutic potential. Curr Pharm Des 2007; 13: 1105–1112. 17430174 10.2174/138161207780618948

[96] Barceló AC Filippini B Pazo JH. The striatum and pain modulation. Cell Mol Neurobiol 2012; 32: 1–12. 21789630 10.1007/s10571-011-9737-7 PMC11498585

