# Supplementary material for: Pharmacological modulation of ventral tegmental area neurons elicits changes in trigeminovascular sensory processing and is accompanied by glycemic changes: Implications for migraine
Source: Cephalalgia. 2022 Oct 18;42(13):1359–74. doi: 10.1177/03331024221110111 (PMC9638709; doi:10.1177/03331024221110111)
Supplement: sj-pdf-1-cep-10.1177_03331024221110111 - Supplemental material for Pharmacological modulation of ventral tegmental area neurons elicits changes in trigeminovascular sensory processing and is accompanied by glycemic changes: Implications for migraine [file sj-pdf-1-cep-10.1177_03331024221110111.pdf]

Pharmacological modulation of ventral tegmental area neurons elicits changes in trigeminovascular sensory processing and is accompanied by glycemic changes: implications for migraine

Margarida Martins-Oliveira[a,b,c], Simon Akerman[d], Philip R. Holland[a], Isaura Tavares[c], Peter J. Goadsby[a,e]

[a] Headache Group, Wolfson Centre for Age-Related Disease, Institute of Psychiatry, Psychology and Neuroscience, King's College London, UK

[b] Department of Nutrition and Metabolism, NOVA Medical School|Faculdade de Ciências Médicas, NMS|FCM,, Universidade Nova de Lisboa; Lisboa, Portugal

[c] Department of Biomedicine, Faculty of Medicine of University of Porto; and Institute of Investigation and Innovation in Health (i3S), University of Porto, Alameda Prof. Hernâni Monteiro, 4200-319 Porto, Portugal

[d] Department of Neural and Pain Sciences, University of Maryland Baltimore, 650 W. Baltimore Street, Baltimore, Maryland, MD 21201, USA

[e] Department of Neurology, University of California, Los Angeles, Los Angeles CA USA

Supplemental material

Supplemental material

Figure S1: Overview of location of TCC recording and VTA^{PBP} microinjection sites.

A, The location of recording sites in the TCC from which recordings of nociceptive neurons, receiving convergent input from the dura mater and facial receptive field, were made. The locations were reconstructed from lesions (black dots) and are located in *laminae* II –V, predominantly in lamina V. **B,** Histological example for the lesion mark (brown lesion as indicated by the arrow) of the recording site in the TCC (lamina V), marked by electrothermolytic lesion (6-10µA for 60 sec). The section was counterstained with cresyl violet. Scale bar, 200 µm. **C,** Representative atlas plate rat brain sections [22] indicating the location of microinjections inside the VTA^{PBP} (black dots) and adjacent to the VTA^{PBP} (black stars). Numbers on atlas plate rat brain sections indicate the distance in millimeters from bregma. **D,** Histological example of microinjection site in the VTA^{PBP}, marked by Chicago Sky Blue and blood (as indicated by the arrow). Section was counterstained with cresyl violet. Scale bar, 200 µm. C1, spinal cord cervical 1; cp, cerebral peduncle; fr, fasciculus retroflexus; IF, interfascicular nucleus; IPF, interpeduncular fossa; ml, medial lemniscus; ML, medial mammillary nucleus, lateral part; MMA, middle meningeal artery; mp, mammillary peduncle; MT, medial terminal nucleus of the accessory optic tract; mtg, mammillotegmental tract; PBP, parabrachial pigmented nucleus of the ventral tegmental area (herein named VTA^{PBP} throughout the text); PN, paranigral nucleus; RLi, rostral linear nucleus of the raphe; RMC, red nucleus, magnocellular part; RPC, red nucleus, parvicellular part; SNCD, substantia nigra, compact part, dorsal tier; SNL, substantia nigra, lateral part; SNR, substantia nigra, reticular part; SuM, supramammillary nucleus; TCC, trigeminocervical complex; TNC, trigeminal

nucleus caudalis; VTAR, ventral tegmental area, rostral part; vtgd, ventral tegmental decussation; VTM, ventral tuberomammillary nucleus.

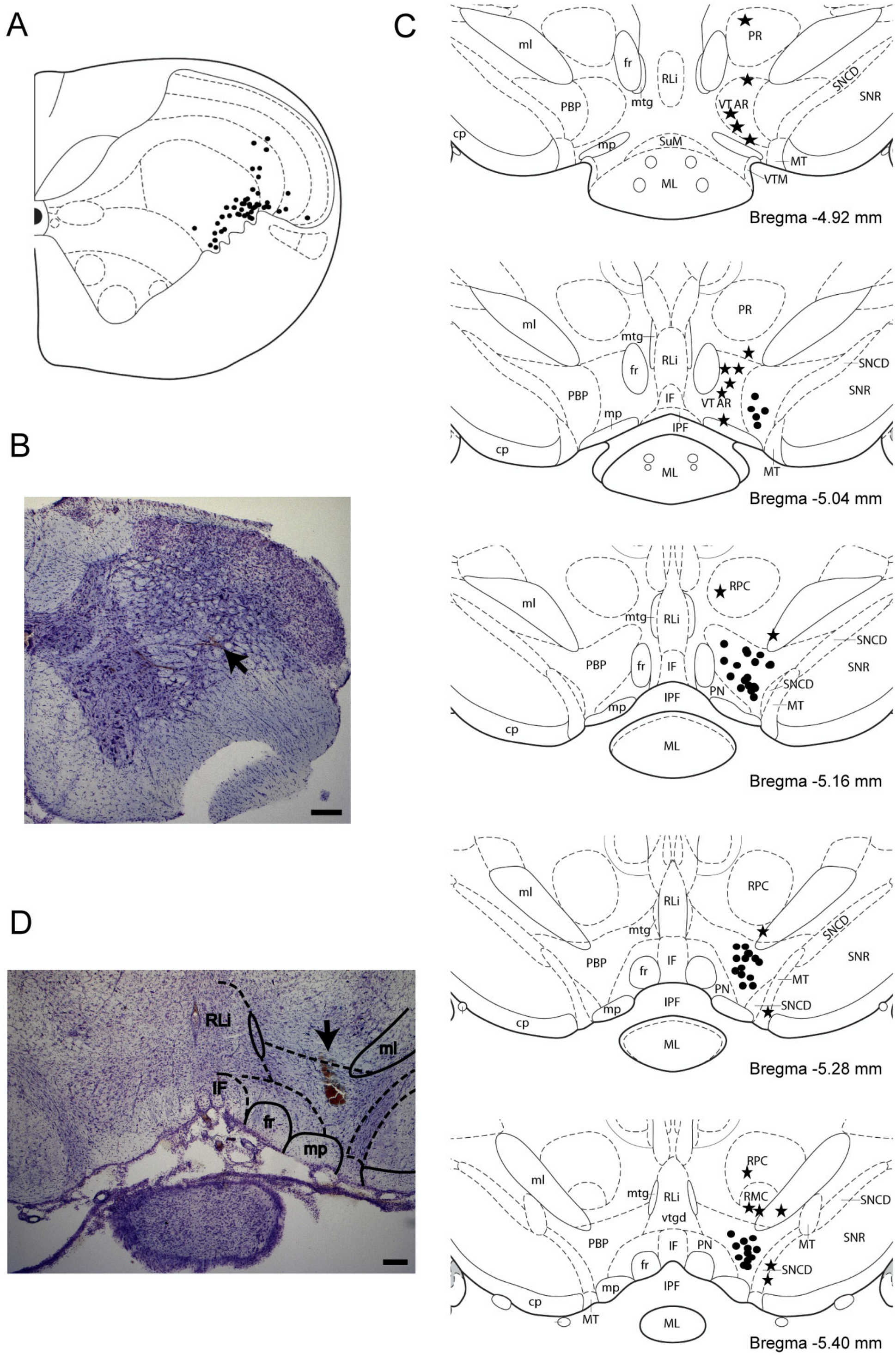

Figure S2: Effects of pharmacological manipulation of VTA^{PBP} on dural-evoked neuronal firing in the trigeminocervical complex (TCC).

A, Poststimulus histograms showing baseline responses to electrical stimulation of the dura mater (left) and the maximum inhibitory effect (right) after VTA^{PBP} microinjection of vehicle control, glutamate and bicuculline.

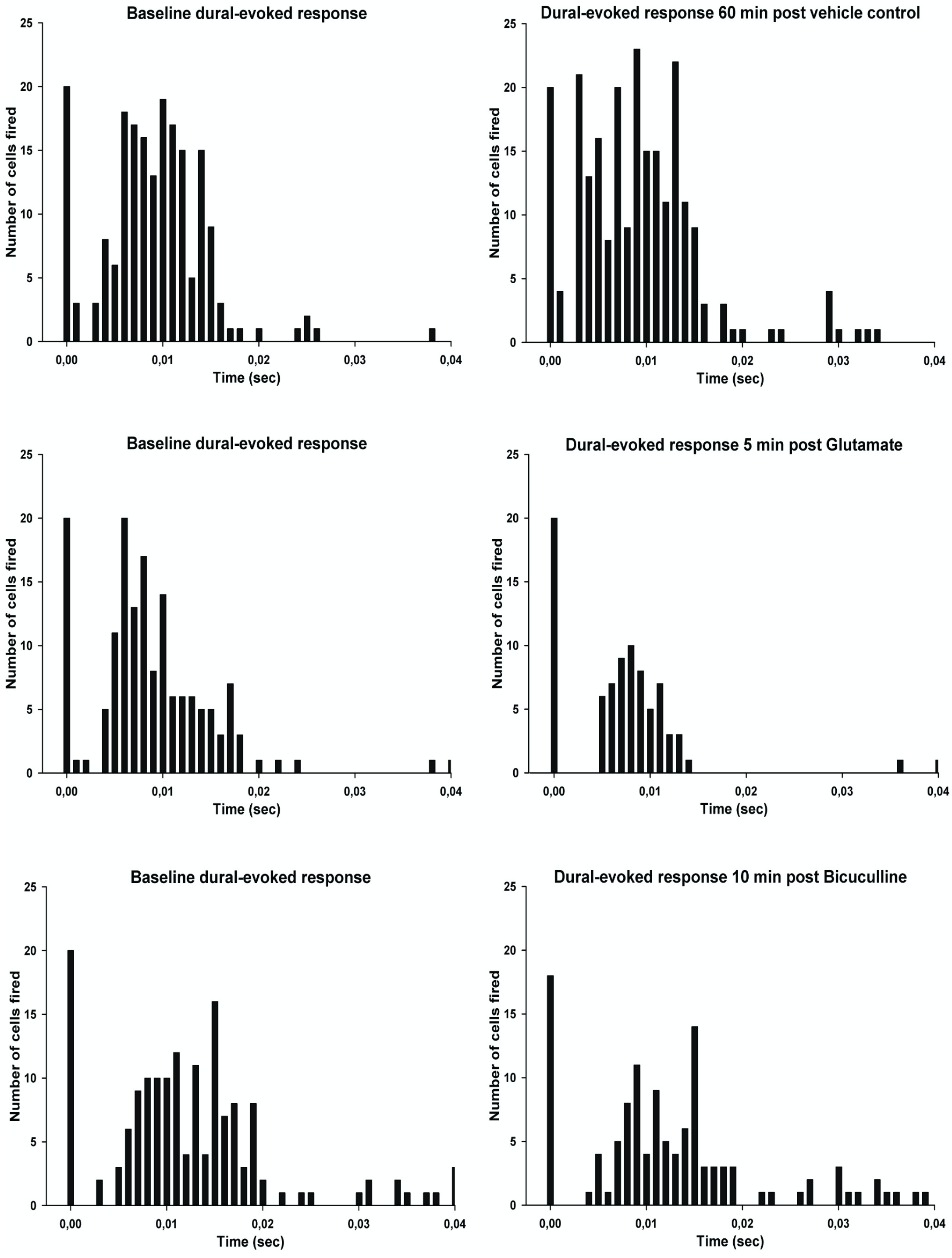

B, Poststimulus histograms showing baseline responses to electrical stimulation of the dura mater (left) and the maximum inhibitory effect (right) after VTA^{PBP} microinjection of naratriptan, PACAP38 and quinpirole.

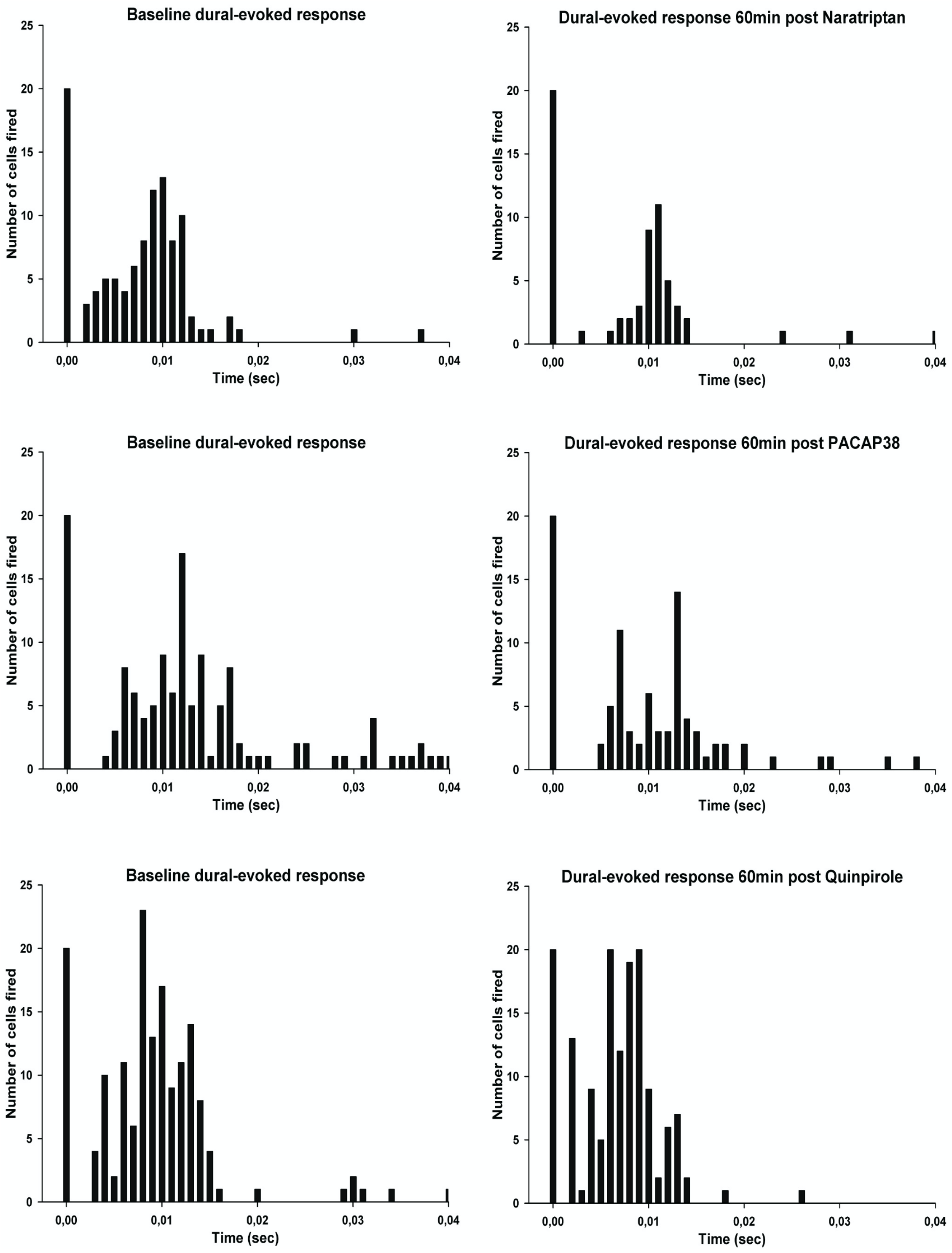

Table S1: Summary of ongoing spontaneous firing rate after 5 and 10 min for each treatment group (Hz). Raw data is presented as MEAN± SEM (min, max rate).

Spontaneous firing rate	5 min post-injection (Hz)	10 min post-injection (Hz)
**Vehicle control (n=12)**	45.0 ± 4.9<br>(20.8, 68.7)	43.9 ± 4.9<br>(20.7, 72.2)
**Glutamate (n=8)**	11.0 ± 1.2<br>(5.8, 16.8)	29.1 ± 5.4<br>(10.5, 52.0)
**Bicuculline (n=11)**	35.7 ± 4.4<br>(11.7, 61.5)	31.2 ± 3.4<br>(11.9, 47.1)
**Naratriptan (n=8)**	16.5 ± 4.9<br>(2.8, 43.6)	15.7 ± 4.5<br>(2.7, 37.9)
**PACAP38 (n=7)**	13.9 ± 3.8<br>(2.5, 27.9)	14.3 ± 3.5<br>(4.1, 28.1)
**Quinpirole (n=8)**	5.3 ± 0.9<br>(3.0, 9.6)	4.4 ± 0.7<br>(2.0, 8.0)

Table S2: Effects of trigeminovascular processing through VTA^{PBP} modulation on blood glucose (BG) levels. Tail vein blood glucose was quantified using a glucometer (in mg/dL) and data was converted into mmol/L (1 mg/dL equals 0.0555 mmol/L). Data is presented as MEAN ± SEM. Maximum responses (%) following VTA^{PBP} microinjection of bicuculline, glutamate, naratriptan, PACAP38, quinpirole and vehicle control. Raw data was used to perform statistical analysis. =NS: not significant; 🡻decreases

	**BG pre-injection (mmol/L)**	**BG 60min post VTA^{PBP} microinjection (mmol/L)**	**Maximum responses (%)**	***p*-value**
**Vehicle control (*n*=7)**	5.01 ± 0.09	4.78 ± 0.10	🡻4%	*p* = 0.188 (NS)
**Bicuculline (*n*=10)**	5.13 ± 0.15	4.56 ± 0.14	🡻11%	*p* = 0.005
**Glutamate (*n*=8)**	5.71 ± 0.15	5.09 ± 0.20	🡻11%	*p* = 0.020
**Naratriptan (*n*=8)**	5.76 ± 0.28	5.03 ± 0.28	🡻12%	*p* = 0.027
**PACAP38 (*n*=7)**	5.93 ± 0.22	4.86 ± 0.37	🡻17%	*p* = 0.034
**Quinpirole (*n*=8)**	5.39 ± 0.24	5.36 ± 0.45	🡻1%	*p* = 0.937 (NS)
